# Author Correction: Longitudinal assessment of tumor development using cancer avatars derived from genetically engineered pluripotent stem cells

**DOI:** 10.1038/s41467-020-15828-2

**Published:** 2020-04-20

**Authors:** Tomoyuki Koga, Isaac A. Chaim, Jorge A. Benitez, Sebastian Markmiller, Alison D. Parisian, Robert F. Hevner, Kristen M. Turner, Florian M. Hessenauer, Matteo D’Antonio, Nam-phuong D. Nguyen, Shahram Saberi, Jianhui Ma, Shunichiro Miki, Antonia D. Boyer, John Ravits, Kelly A. Frazer, Vineet Bafna, Clark C. Chen, Paul S. Mischel, Gene W. Yeo, Frank B. Furnari

**Affiliations:** 1 Ludwig Cancer Research San Diego Branch, 9500 Gilman Dr., CMM-East Room 3055, La Jolla, CA 92093 USA; 2 Department of Neurosurgery, University of Minnesota, 420 Delaware St SE, Minneapolis, MN 55455 USA; 3 Department of Cellular and Molecular Medicine, University of California San Diego, 2880 Torrey Pines Scenic Drive, La Jolla, CA 92093 USA; 4 Department of Pathology, University of California San Diego, 9500 Gilman Dr., La Jolla, CA 92093 USA; 5 Institute for Genomic Medicine, University of California San Diego, 9500 Gilman Dr., Mail Code 0761, La Jolla, CA 92093 USA; 6 Department of Computer Science and Engineering, University of California San Diego, 9500 Gilman Dr., Mail Code 0404, La Jolla, CA 92093 USA; 7 Department of Neuroscience, University of California San Diego, 9500 Gilman Dr., Mail Code 0662, La Jolla, CA 92093 USA; 8 Department of Pediatrics and Rady Children’s Hospital, University of California San Diego, 9500 Gilman Dr., Mail Code 0831, La Jolla, CA 92093 USA

**Keywords:** Cancer models, CNS cancer

Correction to: *Nature Communications* 10.1038/s41467-020-14312-1, published online 28 January 2020.

The original version of this Article contained an error in Fig. 1. The gel image in Fig. 1c was inadvertently taken from Supplementary Fig. 6 instead of Supplementary Fig. 1 that showed the original scan corresponding to Fig. 1c.

This has been corrected in both the PDF and HTML versions of the Article.

