# Supplementary material for: Longitudinal assessment of tumor development using cancer avatars derived from genetically engineered pluripotent stem cells
Source: Nat Commun. 2020 Jan 28;11:550. doi: 10.1038/s41467-020-14312-1 (PMC6987220; doi:10.1038/s41467-020-14312-1)
Supplement: Supplementary file 1 — Supplementary Information [file 41467_2020_14312_MOESM1_ESM.pdf]

Supplementary Figures

Longitudinal assessment of tumor development using cancer avatars derived from genetically engineered pluripotent stem cells

Koga, Chaim *et al*.

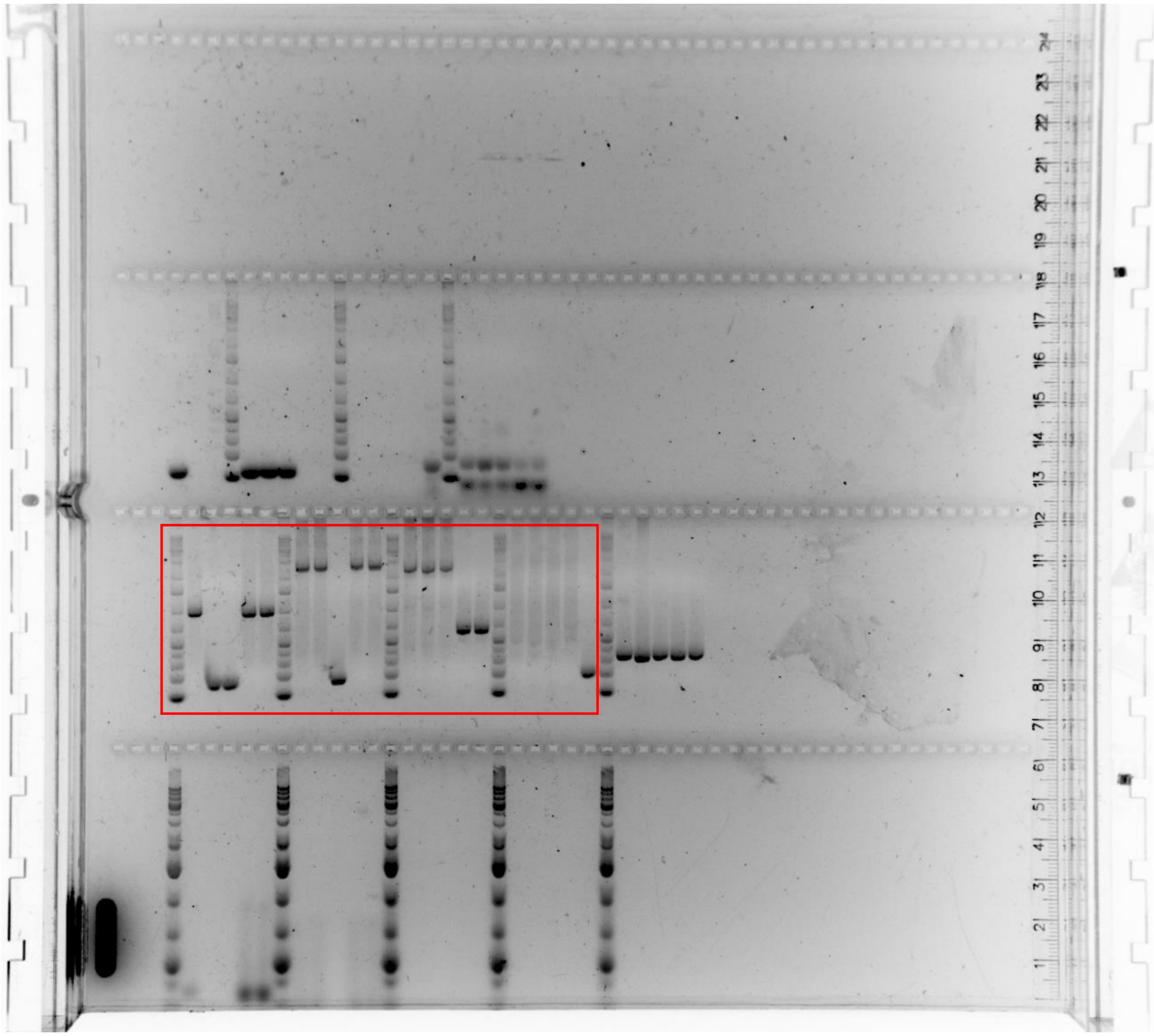

Supplementary Figure 1

An un-cropped image of the agarose gel displayed in Fig. 1c. A red box indicates the area shown in display.

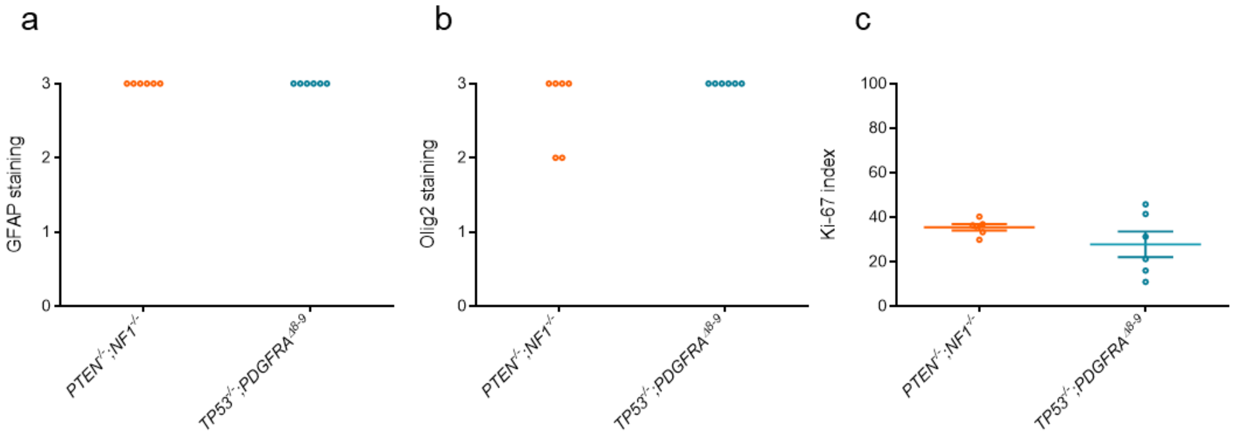

Supplementary Figure 2

iHGG shows high GFAP, Olig2, and Ki-67 positivity. Quantification of IHC staining of iHGGs for GFAP (**a**) and Olig2 (**b**). Data are representative of six replicates, n = 6 slides for each model. **c** Quantification of Ki-67 positivity. Data are representative of six replicates, n = 6 slides for each model. Data are represented as mean ± SEM. Source data are provided as a Source Data file.

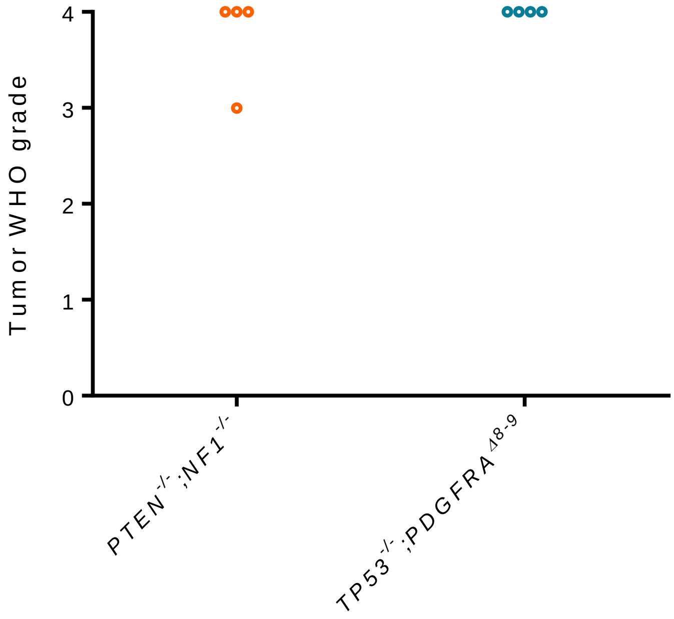

Supplementary Figure 3

iHGG shows features of WHO grade 3 and 4 gliomas. Numbers of iHGG tumors graded as WHO grade 3 and 4 gliomas. Data are representative of four replicates, n = 4 slides for each model. Source data are provided as a Source Data file.

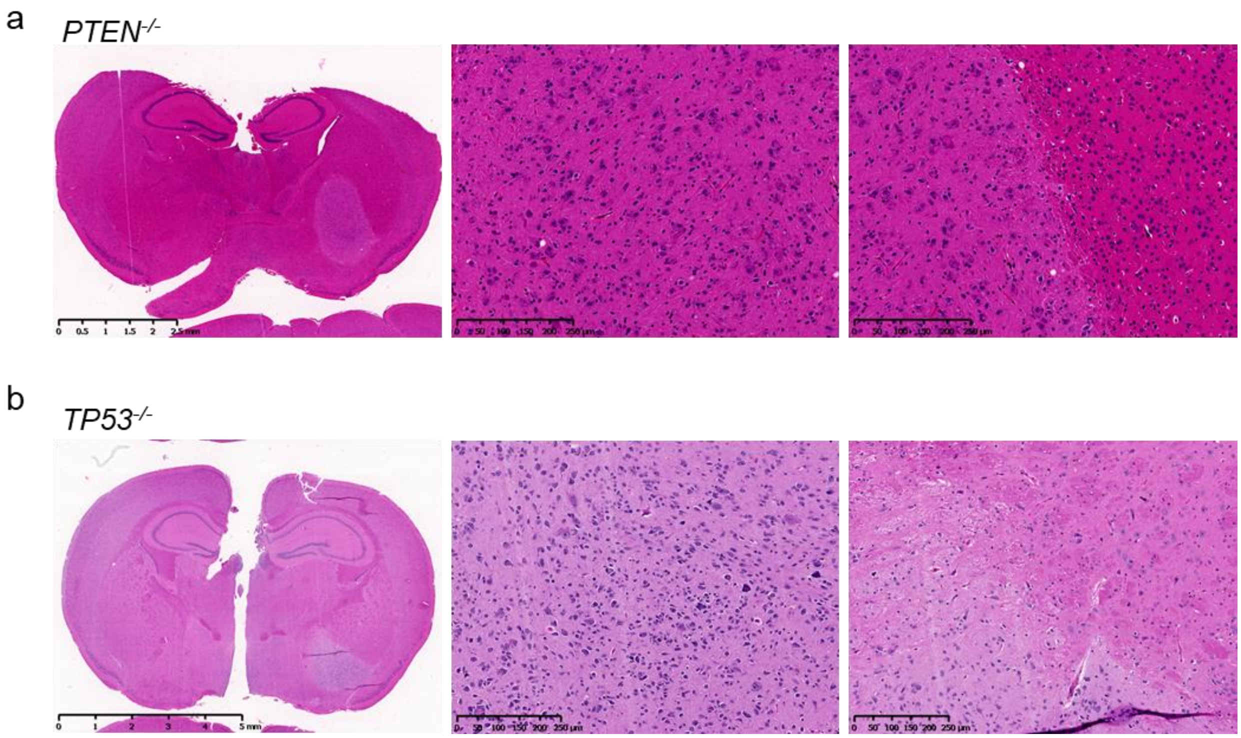

Supplementary Figure 4

PTEN$^{-/-}$ and *TP53*$^{-/-}$ singly edited NPCs do not give rise to tumors. **a** Hematoxylin and eosin staining of mouse brain 9 months after injection of *PTEN*$^{-/-}$ NPCs. (Scale bars in the left, middle and the right panels are 2.5 mm, 250 μm, and 250 μm, respectively.) **b** Hematoxylin and eosin staining of the brain 9 months after injection of *TP53*$^{-/-}$ NPCs. (Scale bars in the left, middle and the right panels are 5 mm, 250 μm, and 250 μm, respectively.)

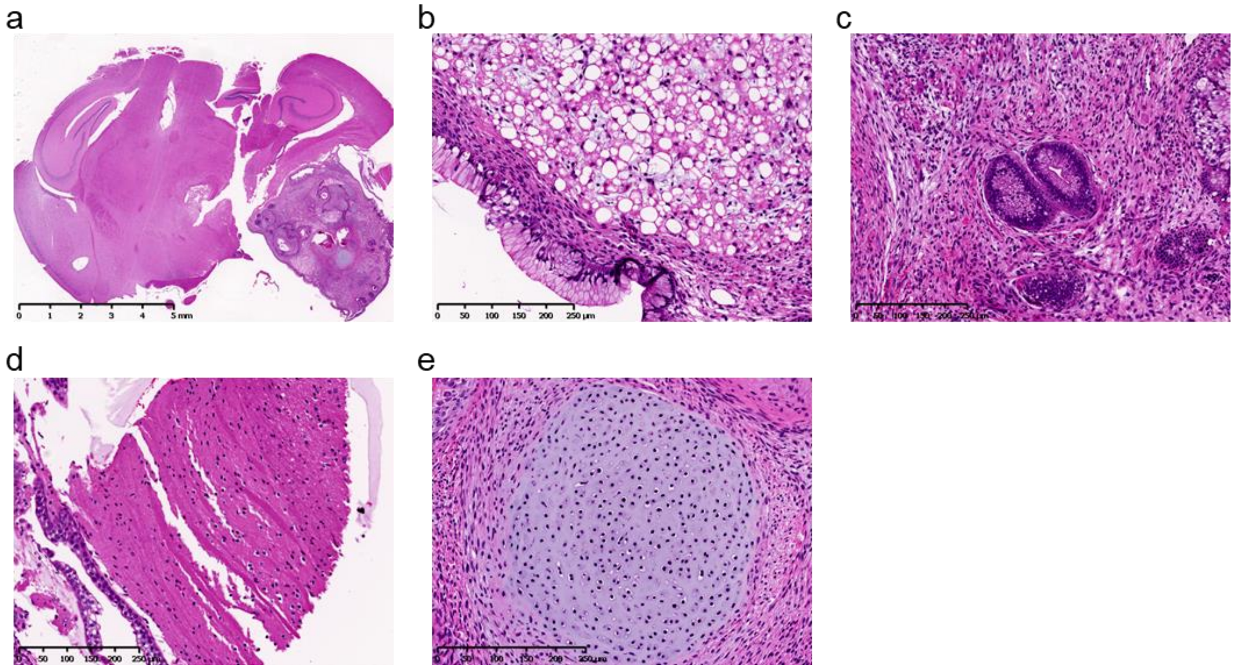

Supplementary Figure 5

Wildtype hiPSCs form teratoma-like tumors in the mouse brain. **a** Hematoxylin and eosin staining of mouse brain post-injection of wildtype hiPSCs. (Scale bar is 5 mm.) **b-e** Hematoxylin and eosin staining of tumors. (Scale bars are 250 µm.) Adipose tissue-like (**b**), gastrointestinal tract-like (**c**), muscle-like (**d**), and cartilage-like tissues (**e**) are observed.

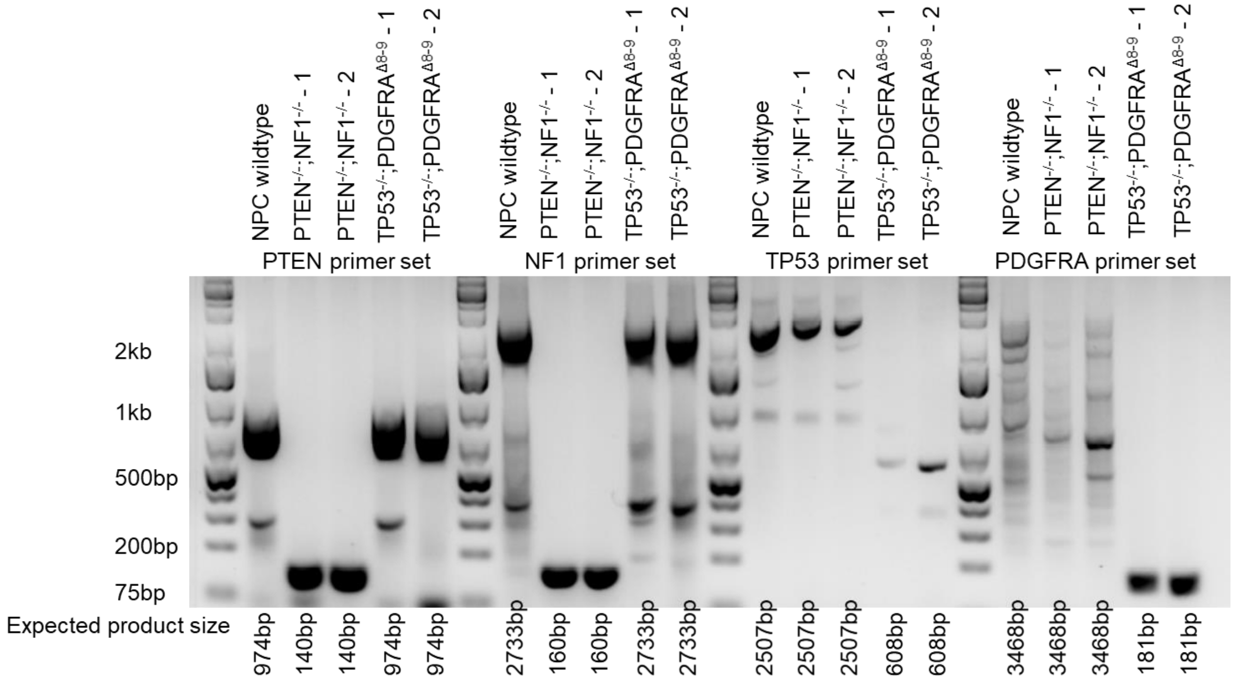

Supplementary Figure 6

iHGG samples have the same genotype as pre-engraftment NPCs. Agarose gel electrophoresis of amplicons from genotyping PCR for each gene in iHGG samples. PCR amplicons from targeted alleles were expected to be 140, 160, 608, and 181 bases for *PTEN*, *NF1*, *TP53*, and *PDGFRA*, respectively.

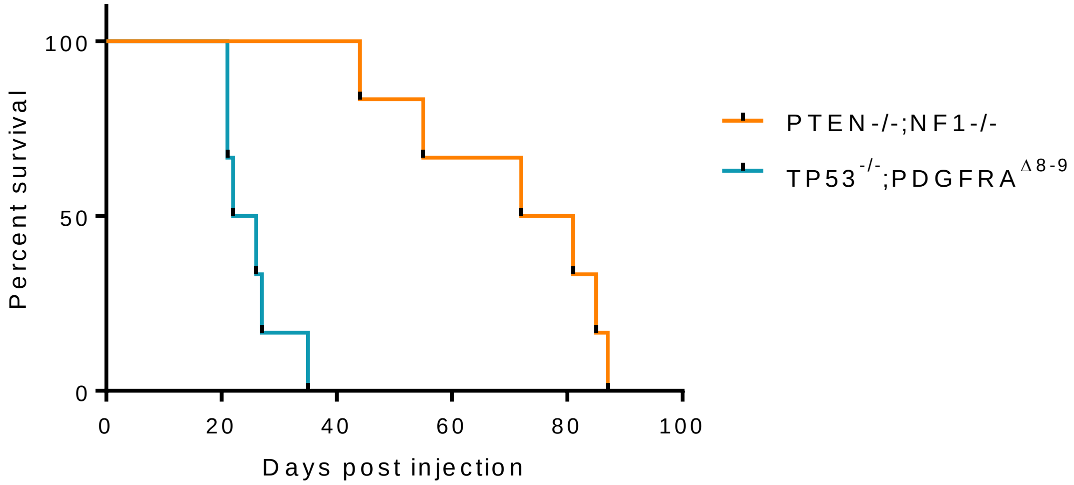

Supplementary Figure 7

Survival of mice with the secondary iHGGs. Kaplan-Meier curves of mice engrafted with the primary iHGGs. Vehicle treated groups from the experiments shown in Fig. 3. Statistical significance was evaluated by the log rank test. Data are representative of six replicates, n = 6 animals for each model. Source data are provided as a Source Data file.

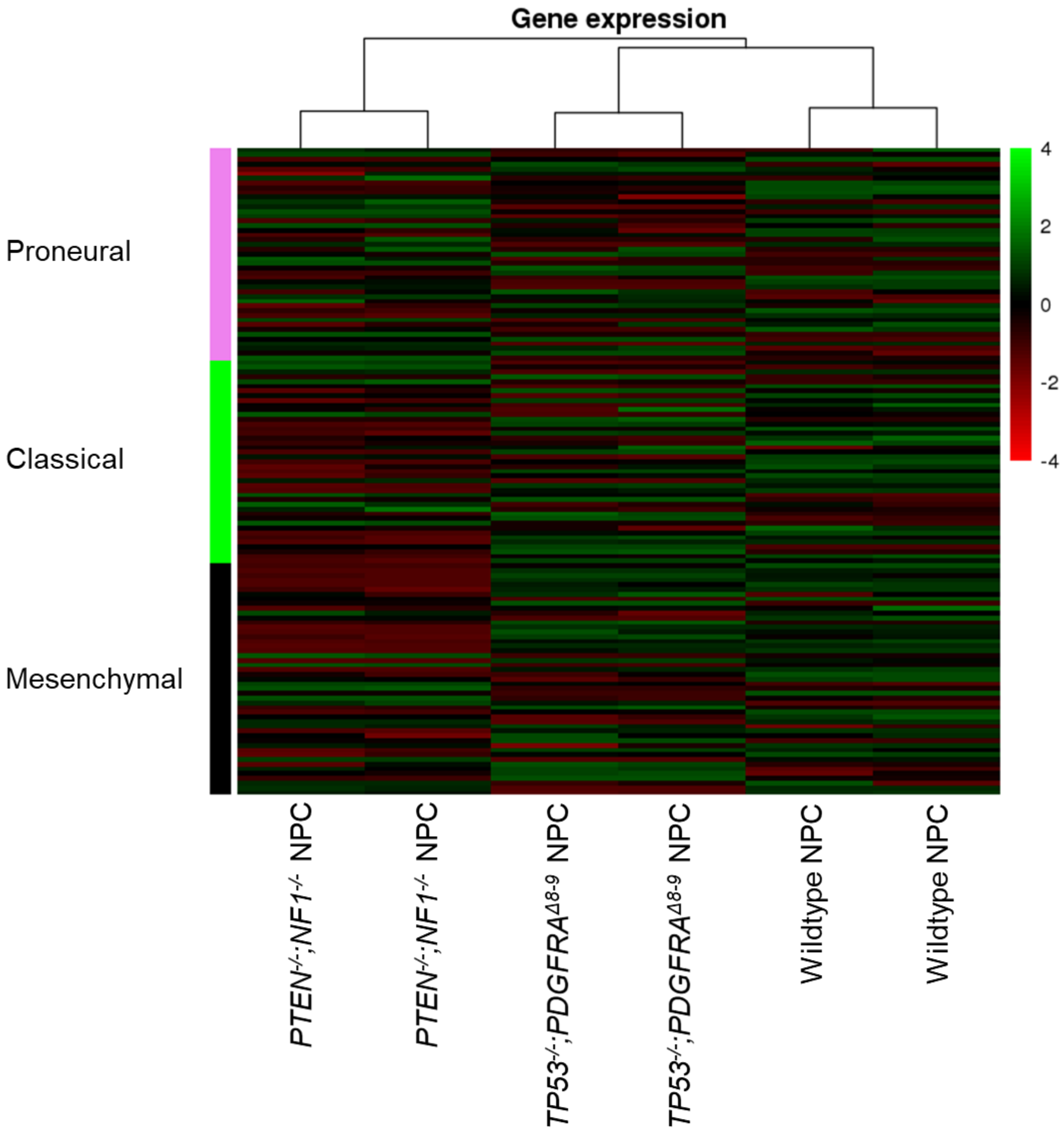

Supplementary Figure 8

NPCs with different genetic modifications lack GBM subtype specific signatures that are observed in each corresponding iHGG. Heatmap for GBM subtype gene transcript levels of wildtype, *TP53*$^{-/-}$;*PDGFRA*$^{\Delta 8\text{-}9}$, and *PTEN*$^{-/-}$;*NF1*$^{-/-}$ NPCs.

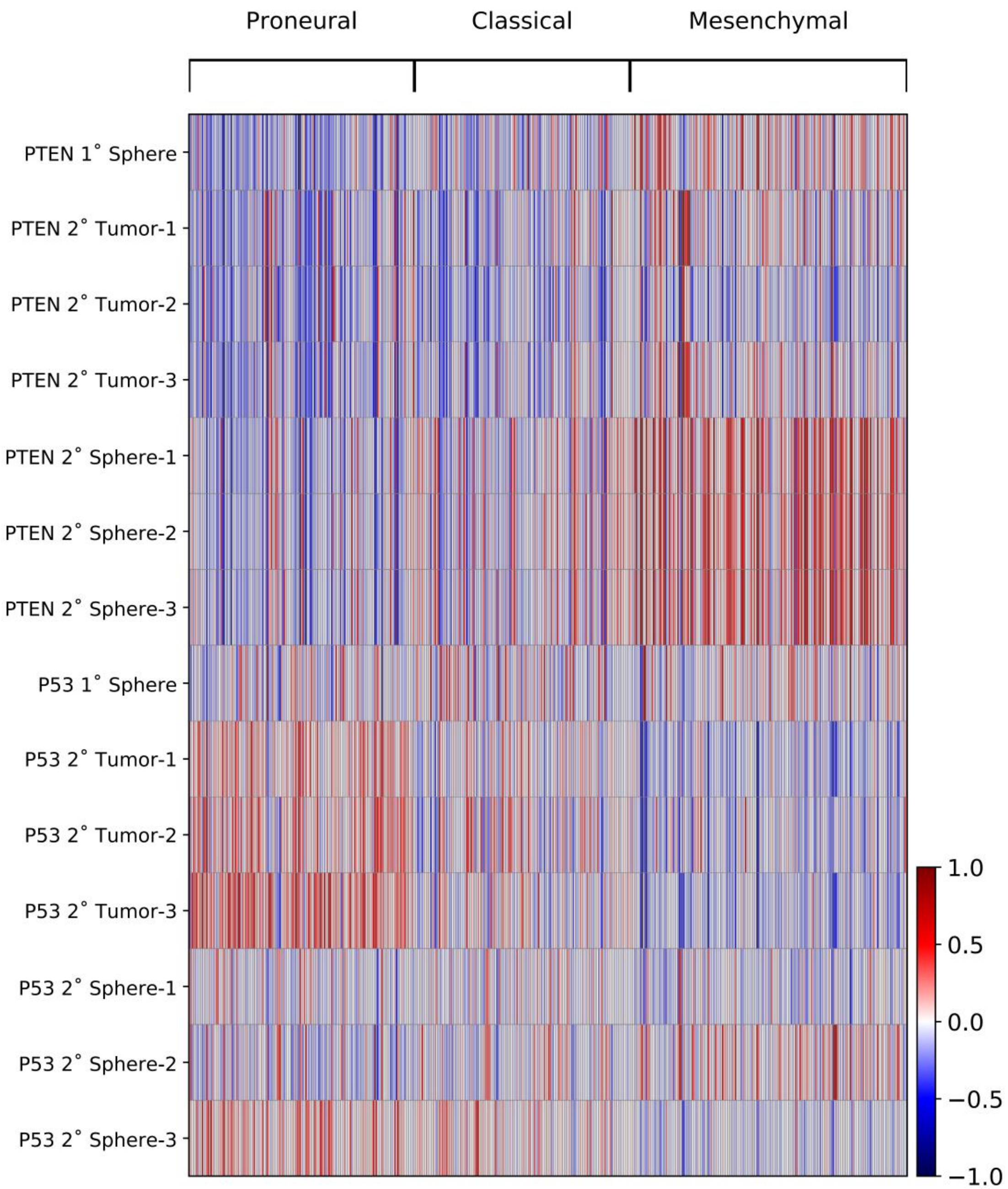

Supplementary Figure 9

iHGG models show inter-sample heterogeneity with characteristic molecular subtype signatures. Heatmaps of average gene expression, per sample, for each TCGA molecular subtype classifier gene.

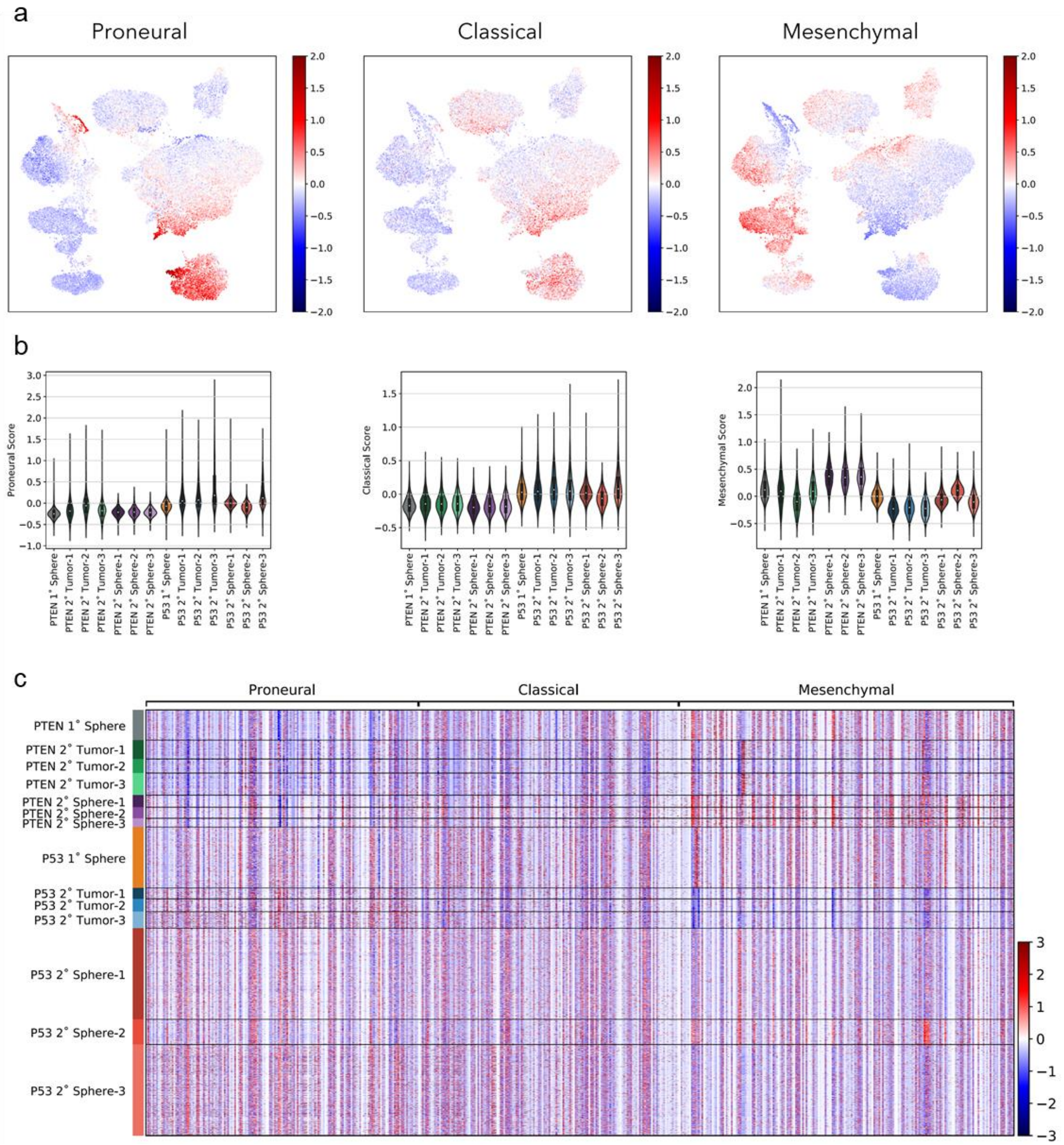

Supplementary Figure 10

iHGG samples present intra-tumor heterogeneity with subpopulations of different GBM molecular subtypes. Subtype scores were calculated for each cell and results overlaid on a UMAP plot (**a**) or summarized as violin plots (**b**). Heatmaps (**c**) of gene expression, for each cell in all samples, for each TCGA molecular subtype classifier gene.

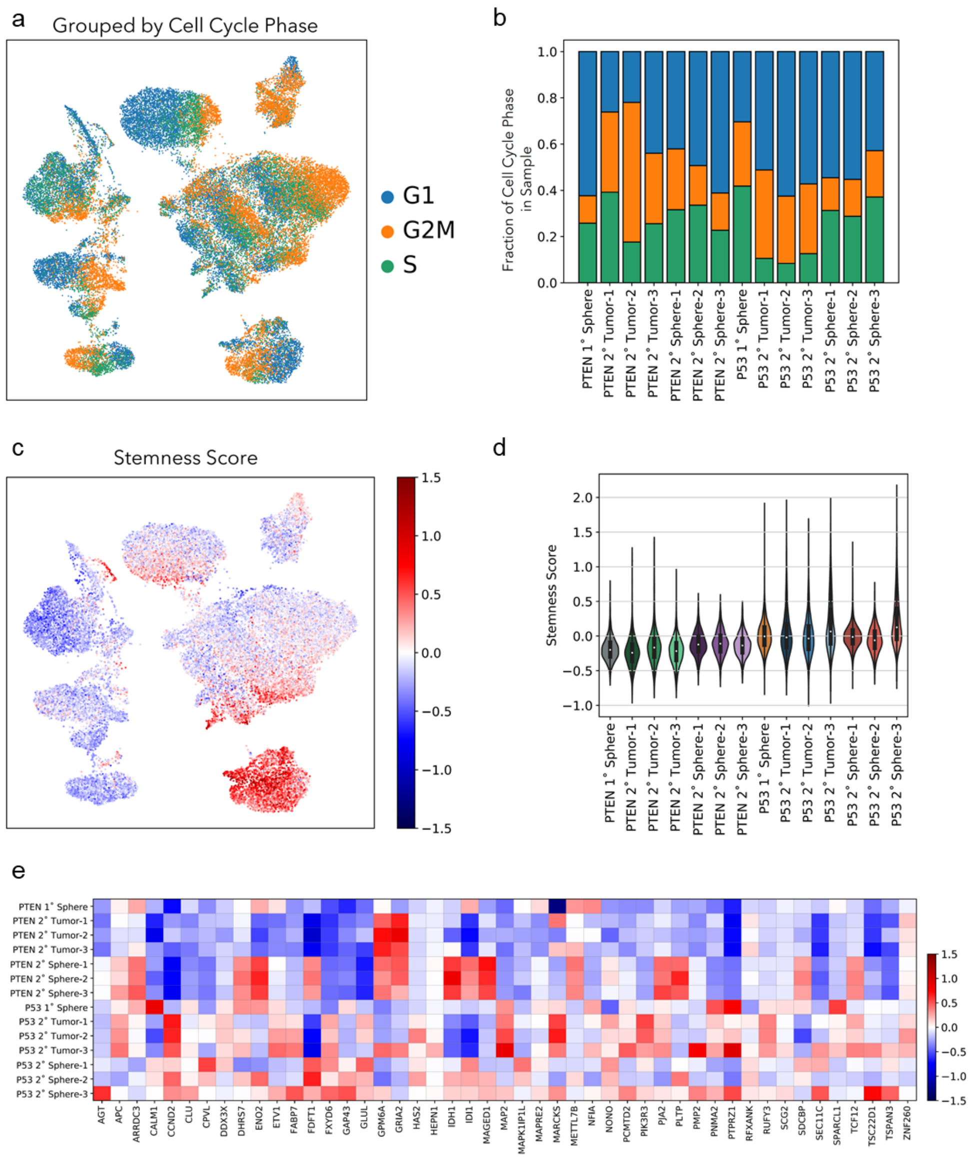

Supplementary Figure 11

Stemness and cell cycle signatures of iHGG models. Cells were categorized based on their cell cycle status (G1, G2M or S), overlaid on a UMAP plot (**a**) and their distribution was calculated for each sample (the color code is same as in a) (**b**). Stemness scores were calculated for each cell and results overlaid on a UMAP plot (**c**) or summarized as violin plots for each sample (**d**). (**e**) Heatmap of stemness-related genes based on average gene expression of individual cells in each sample.

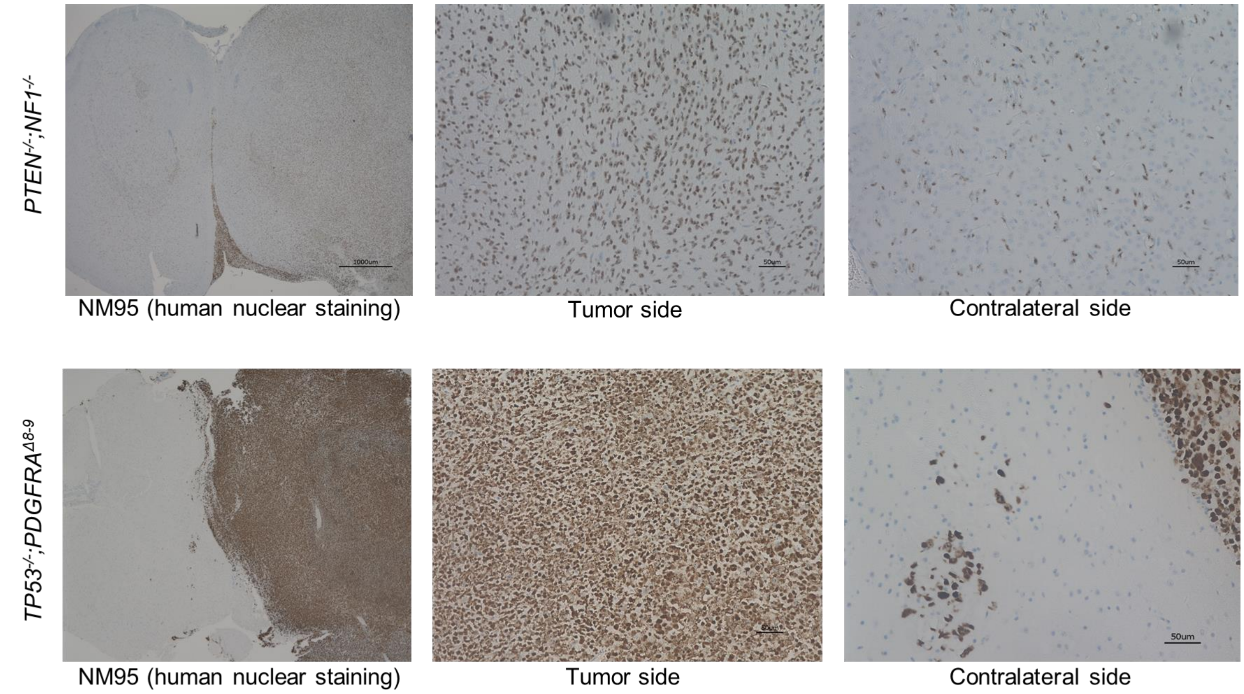

Supplementary Figure 12

PTEN$^{-/-}$;NF1$^{-/-}$ and *TP53$^{-/-}$;PDGFRA$^{\Delta 8\text{-}9}$* iHGGs possess different invasive phenotypes. NM95 human nuclear antibody staining of mouse brains with each iHGG tumor. (Scale bars in the top left, middle, and the right panels are 1000 µm, 50 µm, and 50 µm, respectively. Scale bars in the bottom left, middle, and the right panels are 1000 µm, 50 µm, and 50 µm, respectively.)

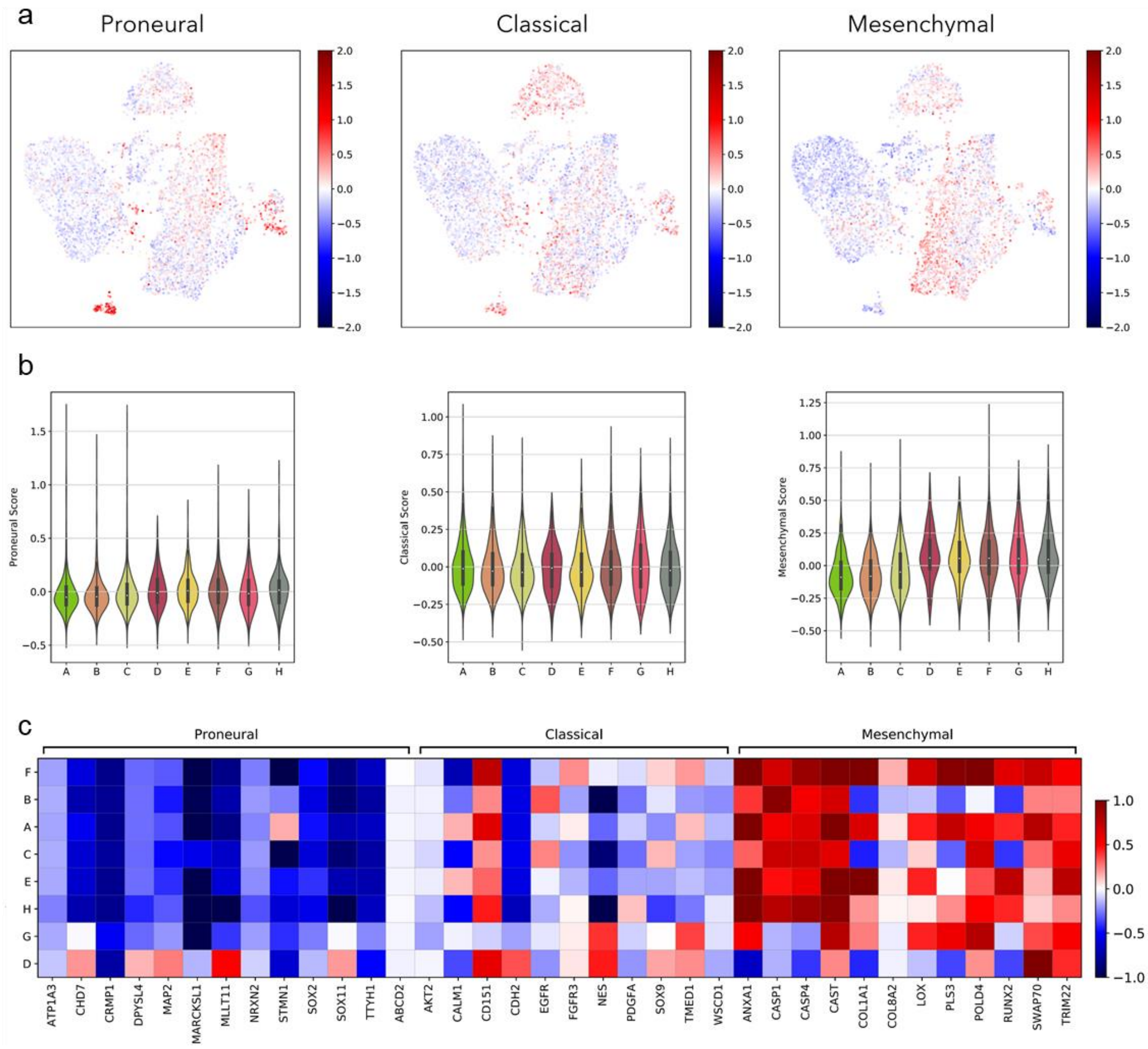

Supplementary Figure 13

iHGG *PTEN*$^{-/-}$;*NF1*$^{-/-}$ spheres present intra-tumor heterogeneity with subpopulations of different GBM molecular subtypes present for each Louvain cluster. Subtype scores were calculated for each cell and results overlaid on a UMAP plot (**a**) or summarized as violin plots (**b**). Heatmap (**c**) of average gene expression, for each Louvain clusters found in iHGG *PTEN*$^{-/-}$;*NF1*$^{-/-}$ spheres , for each TCGA molecular subtype classifier gene. Heatmap scaled values correspond to those calculated for all 14 samples.

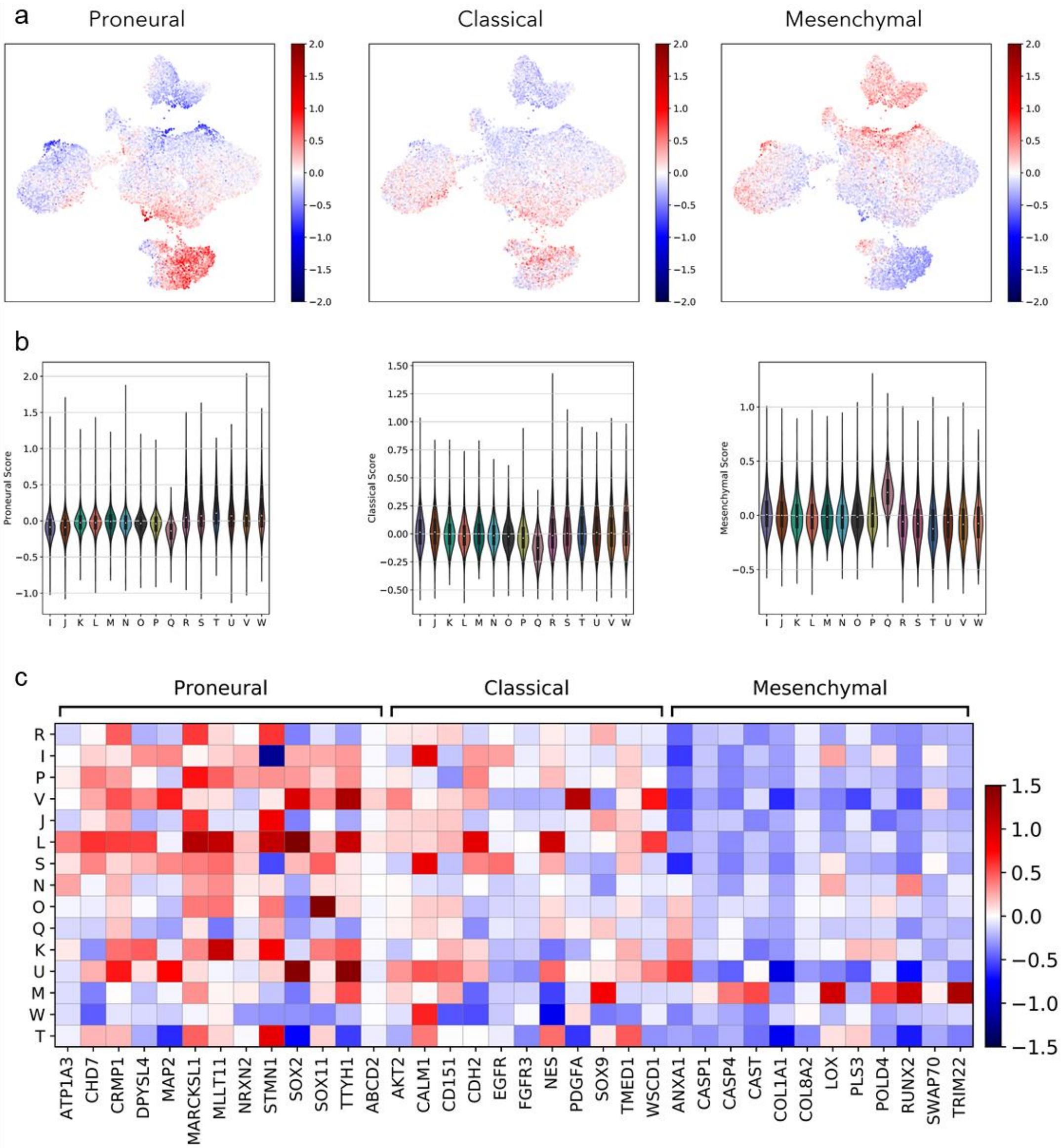

Supplementary Figure 14

iHGG *TP53*$^{-/-}$*;PDGFRA*$^{\Delta 8\text{-}9}$ spheres present intra-tumor heterogeneity with subpopulations of different GBM molecular subtypes present for each Louvain cluster. Subtype scores were calculated for each cell and results overlaid on a UMAP plot (**a**) or summarized as violin plots (**b**). Heatmap (**c**) of average gene expression, for each Louvain clusters found in iHGG *TP53*$^{-/-}$*;PDGFRA*$^{\Delta 8\text{-}9}$ spheres , for each TCGA molecular subtype classifier gene. Heatmap scaled values correspond to those calculated for all 14 samples.

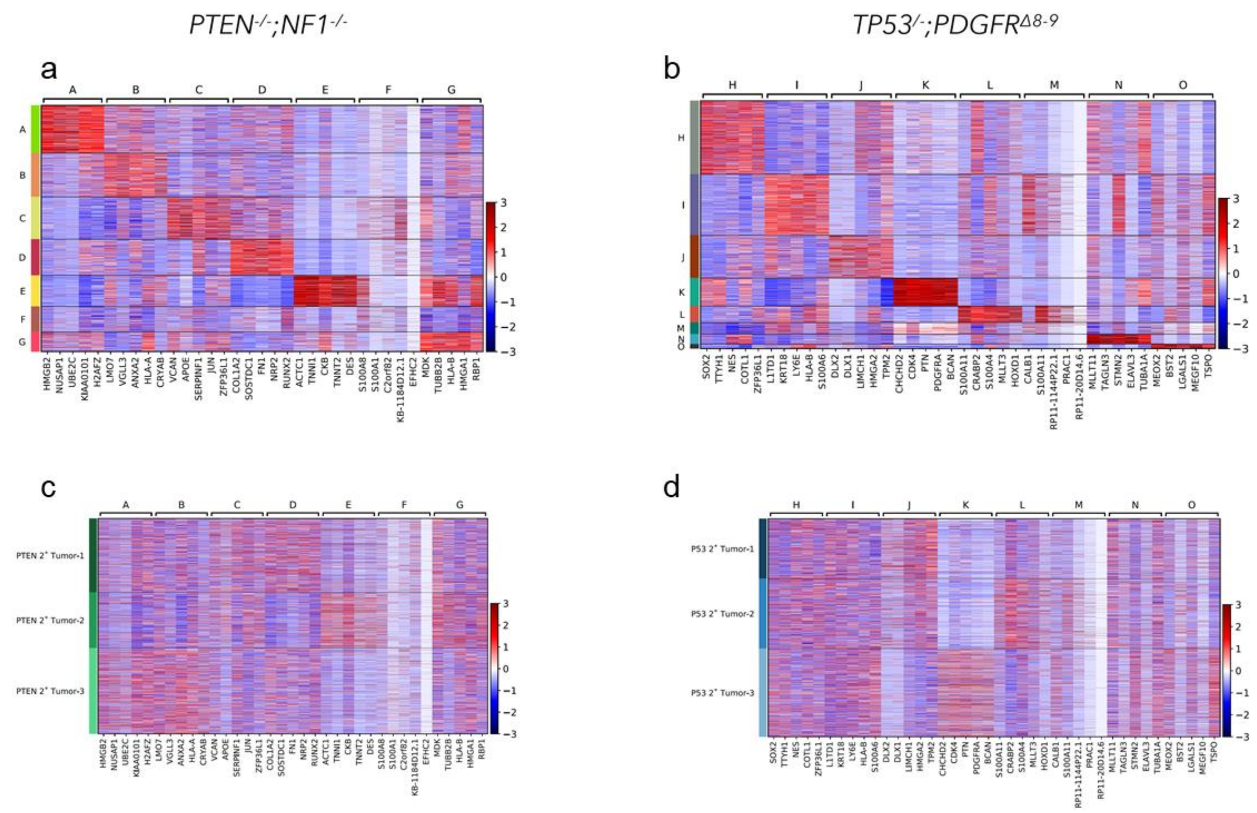

Supplementary Figure 15

Differentially expressed genes driving molecular signatures of tumors' clusters. Heatmaps showing all cells of top-5 Louvain cluster differentially expressed genes, plotted by cluster (**a** and **b**) or by sample identity (**c** and **d**) for *PTEN$^{-/-}$;NF1$^{-/-}$* (**a** and **c**) and *TP53$^{-/-}$;PDGFRA$^{\Delta 8\text{-}9}$* (**b** and **d**).
