# Supplementary material for: Longitudinal assessment of tumor development using cancer avatars derived from genetically engineered pluripotent stem cells
Source: Nat Commun. 2020 Jan 28;11:550. doi: 10.1038/s41467-020-14312-1 (PMC6987220; doi:10.1038/s41467-020-14312-1)
Supplement: Supplementary file 3 — Description of Additional Supplementary Files [file 41467_2020_14312_MOESM3_ESM.pdf]

Description of Additional Supplementary Files

File Name: Supplementary Data 1
Description: Genes used to score and classify: GBM subtypes, cell cycle status and stemness.

File Name: Supplementary Data 2
Description: Differentially expressed genes for all clusters in Figure 5.

File Name: Supplementary Data 3
Description: Gene Ontology analysis extended results for Figure 5.
