# Supplementary material for: Longitudinal assessment of tumor development using cancer avatars derived from genetically engineered pluripotent stem cells
Source: Nat Commun. 2020 Jan 28;11:550. doi: 10.1038/s41467-020-14312-1 (PMC6987220; doi:10.1038/s41467-020-14312-1)
Supplement: Supplementary file 7 — Reporting Summary [file 41467_2020_14312_MOESM7_ESM.pdf]

nature research

Corresponding author(s): Frank B. Furnari, PhD and Gene W. Yeo, PhD

Last updated by author(s): Dec 11, 2019

Reporting Summary

Nature Research wishes to improve the reproducibility of the work that we publish. This form provides structure for consistency and transparency in reporting. For further information on Nature Research policies, see Authors & Referees and the Editorial Policy Checklist.

Statistics

For all statistical analyses, confirm that the following items are present in the figure legend, table legend, main text, or Methods section.

n/a	Confirmed	
☐	☒	The exact sample size (*n*) for each experimental group/condition, given as a discrete number and unit of measurement
☐	☒	A statement on whether measurements were taken from distinct samples or whether the same sample was measured repeatedly
☐	☒	The statistical test(s) used AND whether they are one- or two-sided<br>*Only common tests should be described solely by name; describe more complex techniques in the Methods section.*
☐	☒	A description of all covariates tested
☐	☒	A description of any assumptions or corrections, such as tests of normality and adjustment for multiple comparisons
☐	☒	A full description of the statistical parameters including central tendency (e.g. means) or other basic estimates (e.g. regression coefficient) AND variation (e.g. standard deviation) or associated estimates of uncertainty (e.g. confidence intervals)
☐	☒	For null hypothesis testing, the test statistic (e.g. *F*, *t*, *r*) with confidence intervals, effect sizes, degrees of freedom and *P* value noted<br>*Give P values as exact values whenever suitable.*
☒	☐	For Bayesian analysis, information on the choice of priors and Markov chain Monte Carlo settings
☒	☐	For hierarchical and complex designs, identification of the appropriate level for tests and full reporting of outcomes
☒	☐	Estimates of effect sizes (e.g. Cohen's *d*, Pearson's *r*), indicating how they were calculated

Our web collection on statistics for biologists contains articles on many of the points above.

Software and code

Policy information about availability of computer code

Data collection	No software was used for data collection.
Data analysis	GraphPad Prism 6 software was used for statistical analyses.<br>For single cell data analysis, we used the publicly available Cell Ranger 2.1.1 pipeline (10X Genomics), and the Scanpy v1.3.3 package.

For manuscripts utilizing custom algorithms or software that are central to the research but not yet described in published literature, software must be made available to editors/reviewers. We strongly encourage code deposition in a community repository (e.g. GitHub). See the Nature Research guidelines for submitting code & software for further information.

Data

Policy information about availability of data

All manuscripts must include a data availability statement. This statement should provide the following information, where applicable:

- Accession codes, unique identifiers, or web links for publicly available datasets
- A list of figures that have associated raw data
- A description of any restrictions on data availability

RNA-seq data and single-cell RNA-seq data that support the findings of this study (Fig. 4, 5, and 7, Supplementary Fig. 8-11, and 13-15) have been deposited at the Gene Expression Omnibus, accession number pending.

Field-specific reporting

Please select the one below that is the best fit for your research. If you are not sure, read the appropriate sections before making your selection.

☒ Life sciences ☐ Behavioural & social sciences ☐ Ecological, evolutionary & environmental sciences

For a reference copy of the document with all sections, see nature.com/documents/nr-reporting-summary-flat.pdf

Life sciences study design

All studies must disclose on these points even when the disclosure is negative.

Sample size	A total of 14 samples were collected and profiled by single-cell RNA-seq. These consisted of two sets of seven, one for each genotype studied (PTEN-/-;NF1-/- and TP53-/-;PDGFRAΔ8-9). For each, one sample corresponded to a primary sphere, three to secondary tumors derived from the primary sphere and three secondary spheres derived from each of the secondary tumors.
Data exclusions	Droplets excluded by default CellRanger pipeline parameters, were excluded from analysis. All genes that were not detected in at least 20 single cells were discarded. Cells with fewer than 600 or more than 8,000 expressed genes as well as cells with more than 80,000 transcripts or 0.1% mitochondrial expressed genes were removed from the analysis. For the different sample subset combination analysis filtering steps were the same with the exception of specific gene and transcript thresholds for which cells were removed: PTEN-/-;NF1-/- spheres (fewer than 600 or more than 7000 expressed genes and more than 50,000 transcripts), PTEN-/-;NF1-/- tumors (fewer than 600 or more than 8,000 expressed genes and more than 80,000 transcripts), TP53-/-;PDGFRAΔ8-9 spheres (fewer than 600 or more than 7,000 expressed genes and more than 70,000 transcripts), TP53-/-;PDGFRAΔ8-9 tumors (fewer than 600 or more than 6,000 expressed genes and more than 40,000). All other cells were included in the analysis.
Replication	The experiments were performed at least for three replicates.
Randomization	For the drug treatment experiments, female Nod/Scid mice injected with tumor cells were randomized before being allocated to respective cages.
Blinding	Investigators were not blinded to either allocation during experiments or to data analyses.

Reporting for specific materials, systems and methods

We require information from authors about some types of materials, experimental systems and methods used in many studies. Here, indicate whether each material, system or method listed is relevant to your study. If you are not sure if a list item applies to your research, read the appropriate section before selecting a response.

Materials & experimental systems

n/a	Involved in the study	
☐	☒	Antibodies
☐	☒	Eukaryotic cell lines
☒	☐	Palaeontology
☐	☒	Animals and other organisms
☒	☐	Human research participants
☒	☐	Clinical data

Methods

n/a	Involved in the study	
☒	☐	ChIP-seq
☒	☐	Flow cytometry
☒	☐	MRI-based neuroimaging

Antibodies

Antibodies used	The following antibodies were used: PE-Conjugated Anti-Human HLA-A,B,C (BioLegend, Cat#311406, Lot#B200224), GFAP (Dako, Cat#Z0334), Olig2 (Sigma, Cat#HPA003254, Lot#A60686), Ki-67 (Abcam, Cat#21700, Lot#GR322572-1), Anti-Human Nucleoli Antibody (Abcam, Cat#190710). All descriptions about antibodies including manufacturer are noted in the method section of the manuscript.
Validation	Validation of each primary antibody for the species and application, were based on manufacturer's statement available on their respective company website.

Eukaryotic cell lines

Policy information about cell lines

Cell line source(s)	CV-hiPS-B line was a gift from Lawrence S. B. Goldstein (University of California at San Diego).
Authentication	The CV-hiPS-B line was originally described and characterized, including exome-sequencing in Gore et al., Nature. 2011 Mar

Authentication	3;471(7336):63-7. doi:10.1038/nature09805 (https://www.ncbi.nlm.nih.gov/pubmed/21368825) and therefore was not authenticated.
Mycoplasma contamination	All cell lines used were tested for mycoplasma contamination. All tested negative.
Commonly misidentified lines (See ICLAC register)	No.

Animals and other organisms

Policy information about studies involving animals; ARRIVE guidelines recommended for reporting animal research

Laboratory animals	Five-six-week-old female Nod/Scid mice and athymic nu/nu mice were purchased from Charles River Laboratories.
Wild animals	Did not involve wild animals.
Field-collected samples	Did not involve samples collected from the field.
Ethics oversight	UCSD Animal Care Program.

Note that full information on the approval of the study protocol must also be provided in the manuscript.
